# A Mobile Platform Enables Unprecedented Sanitation Uptake in Zambia

**DOI:** 10.1371/journal.pntd.0005131

**Published:** 2017-01-12

**Authors:** Laurie Markle, Abel Maganani, Oswell Katooka, Amy Tiwari, Nicolas Osbert, David A. Larsen, Benjamin Winters

**Affiliations:** 1 Akros, Inc., Lusaka, Zambia; 2 Ministry of Local Government and Housing, Lusaka, Zambia; 3 UNICEF Water and Sanitation unit, Lusaka, Zambia; 4 University of Montana School of Public and Community Health Sciences, Missoula, Montana, United States of America; Oxford University Clinical Research Unit, VIET NAM

## Overview

Today, diarrhea remains the world’s second leading cause of death and is the leading cause of malnutrition and stunting in children under five. Many cases of diarrhea in developing countries can be prevented through access to sanitation and water. In fact, water, sanitation, and hygiene reduce diarrhea risk by an estimated 30%. When Kamal Kar published his first paper on Community-Led Total Sanitation (CLTS) in 2003, 43% of sub-Saharan Africa’s rural population was practicing open defecation. Initially taking flight in Asia, the CLTS approach was a novel shift in sanitation and hygiene improvement interventions from providing individual, household latrine subsidies to propelling subsidy-free, community-driven behavior change. Kar argues that CLTS is effective due to its focus on demand creation. By illustrating the communal costs of open defecation (e.g., that one person’s bad habits result in another person’s bad health), the CLTS program affects a communal resolve, i.e., “demand,” for change. Akros, in partnership with Zambia’s Ministry of Local Government and Housing (MLGH) and UNICEF, layered a unique mobile-to-web application over traditional CLTS delivery methods, resulting in an innovative service delivery and monitoring system dubbed “CLTS M2W.” CLTS M2W uses mobile phones, automated data feedback loops, and engagement of traditional leaders to provide communities with the ability to clearly see their progress towards sanitation goals. CLTS M2W paved the way for unprecedented CLTS uptake in Zambia, facilitating the creation of over 1,500,000 new users of sanitation in 18 months. In short, CLTS creates the demand, and CLTS M2W creates the critical transparency necessary to drive sustained behavior change.

## Challenge

**Fig 1:**
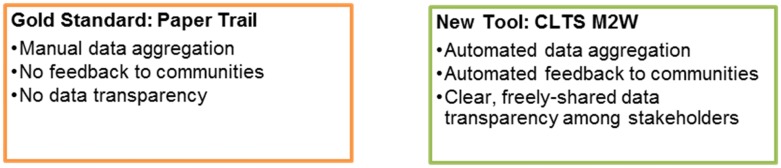
Comparison of previous reporting pathway to new M2W platform.

Today, diarrhea remains the world’s second leading cause of death and is the leading cause of malnutrition and stunting in children under five [1]. Many cases of diarrhea, and over 80% of diseases in developing countries, can be prevented through access to sanitation and water [2]. In fact, water, sanitation, and hygiene reduce diarrhea risk by an estimated 30% [3,4]. In addition to diarrhea, poor sanitation and hygiene have been shown to increase the prevalence of hookworm, *Trichuris trichiura*, *Entamoeba hartmanni*, *Endolimax nana*, and *Blastocystis hominis* infections [5]. When Kamal Kar published his first paper on Community-Led Total Sanitation (CLTS) in 2003 [6], 43% of sub-Saharan Africa’s rural population was practicing open defecation. Initially taking flight in Asia, the CLTS approach was a novel shift in sanitation and hygiene improvement interventions from providing individual, household latrine subsidies to propelling subsidy-free, community-driven behavior change. Kar argues that CLTS is effective due to its focus on demand creation [7]. By illustrating the communal costs of open defecation (e.g., that one person's bad habits result in another person's bad health), the CLTS program affects a communal resolve, i.e., “demand,” for change. Akros, in partnership with Zambia’s MLGH and UNICEF, layered a unique mobile-to-web application (M2W) over traditional CLTS delivery methods, resulting in an innovative service delivery and monitoring system dubbed “CLTS M2W” [8]. CLTS M2W uses mobile phones, automated data feedback loops, and engagement of traditional leaders to provide communities with the ability to clearly see their progress towards sanitation goals. CLTS M2W paved the way for unprecedented CLTS uptake in Zambia, facilitating the creation of over 1,500,000 new users of sanitation over 18 months. In short, CLTS creates the demand, and CLTS M2W creates the critical transparency necessary to drive sustained behavior change. See Fig 1 below for a comparison of traditional CLTS and CLTS M2W reporting models.

## Community Level Feedback

**Fig 2:**
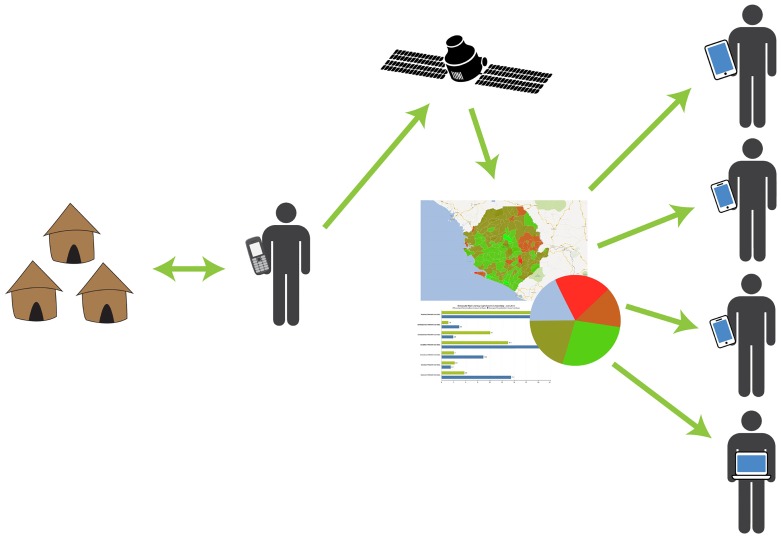
Mobile-to-web reporting pathway.

Using District Health Information System 2 (DHIS2), a cloud-based data aggregation program, and a mobile-to-web surveillance protocol, feedback loops were designed to provide sustainable support and drive competition within the reporting hierarchy [8,9]. Village-level Sanitation Action Groups collect monthly household population, water access, and latrine adequacy data on paper forms. A volunteer Community Champion (CC) collects the paper forms from ten villages each month and submits a village-level aggregate report through a mobile phone via a Java-based application. Designed into the program are Short Message Service (SMS) reminders to CCs to submit monthly village-level sanitation reports, notifications of data validation errors, and notices of sanitation milestone achievements, which promote the sustainability of the M2W system. Reporting rates can be automatically transmitted to volunteer supervisors. District, provincial, and central government personnel receive real-time access to online dashboards providing quick overviews of performance within their constituency and among their peers, as well as monthly automated reports on performance, reporting rates, and data quality, which are circulated throughout the government. Fig 2 below illustrates the CLTS M2W reporting and feedback schematic. This approach has not only driven a data feedback cycle, which improves data quality, but has also established a cost-effective, sustainable solution to CLTS monitoring and evaluation that requires minimal third party investment. According to an internal UNICEF estimate, across Zambia’s non-M2W districts in 2014, the average cost per new sanitation user reached was US$2.50; in M2W districts, new sanitation users were reached at an average unit cost of US$1.65.

## Understanding Community Decision-Making

**Fig 3:**
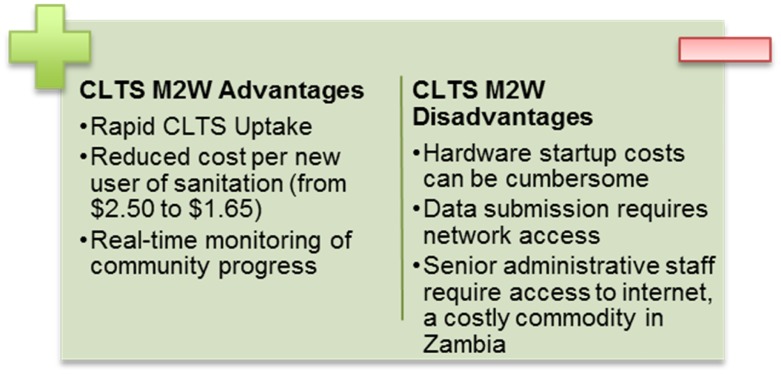
Advantages and disadvantages of mobile-to-web CLTS.

Of the feedback loops designed, the most transformative has been the empowerment of traditional leaders, the key agents of behavior change in rural Zambia. During chiefdom orientations on CLTS M2W, village headmen gather before their Chiefs and are provided the sanitation status of their village compared to neighboring villages. The collective chiefdom then commits to a deadline to claim Open Defecation Free (ODF) status, in which each household has a latrine that meets minimum adequacy standards. To continue to drive pressure following orientations, Chiefs receive tablets outfitted with a custom application. The visualizer application pulls real-time reports from DHIS2 to provide chiefdom-level performance data through maps, graphs, and charts in an easy-to-navigate, simple format. Providing Chiefs with data on village-level performance allows them to make judicious use of limited fuel and monitoring resources and continue to place pressure on CCs to submit reports. This and other salient benefits of CLTS M2W are noted in Fig 3 below.

The Chief’s application, along with the other novel feedback loops, supports a culture of awareness and transparency: neighbors, superiors, and Chiefs are all witness to village-level and CC performance. This act of taking previously private activities (i.e., open defecation) and making them publicly known further motivates behavior change. The M2W protocol also propels rapid reporting throughout administrative hierarchies among stakeholder ministries and, by backing a carefully selected set of indicators with evidence, provides decision-makers with the ability to make data-driven decisions. Together, this culture of awareness, interministerial accountability, and a set of robust data validation rules within DHIS2 enables a rapid, cost-effective, and sustainable means of data verification.

## Creating a Culture of Surveillance

**Fig 4:**
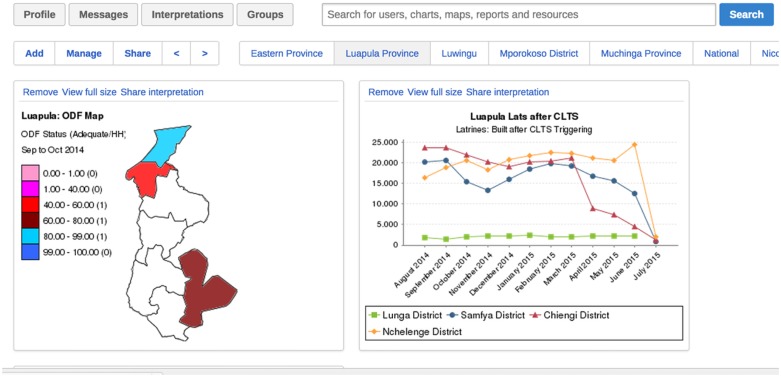
Sample provincial-level dashboard.

Such verification procedures have become part and parcel of the CLTS program because of their effectiveness. For instance, through the technical support afforded by M2W, CLTS-implementing partners have been able to quickly address the dynamicity of the ODF status. For example, with the data provided in the M2W platform, MLGH was able to identify hand washing station coverage as the most challenging ODF component to attain in a community and, due to the requirements of water procurement, container quality, and access to soap, the most challenging to sustain. Armed with such knowledge, agents of community behavior change are able to target their interventions and activities through the same platform that is creating a culture of accountability amongst their communities. In this way, the CLTS M2W platform supports sustainability by enabling community buy-in, building capacity for critical thinking, and empowering leaders with real-time data, leading to improved intervention outcomes. A sample dashboard, facilitating access to this real-time data, is provided in Fig 4 below.

## Way Forward

Escalation of the CLTS M2W platform is not without challenges. Building capacity among rural populations to engage technology and interpret data is time-consuming. Mobile phones require access to cell networks, senior administrators require costly internet access, and initial hardware expenses can be burdensome. Solving these challenges will require integration of the M2W platform across multiple sectors [10], but challenges integrating water and sanitation data with health indicator data (collected by the Ministry of Health) remain. Because health data, such as diarrhea prevalence, is the mandate of the Ministry of Health and rural water and sanitation data the mandate of MLGH, there’s limited political will to ensure that health outcomes are measured as part of the routine reporting protocol. Additional studies across countries to ensure that the success of Zambia’s M2W CLTS program is not unique are required.

M2W CLTS is currently operating across 49 of Zambia’s 76 rural districts. Over the next year, M2W CLTS will be scaled up to include 100% of Zambia’s rural districts, will incorporate School-Led Total Sanitation into the surveillance platform, and will expand to include monitoring of water access and facial cleanliness with the aim of better targeting trachoma prevention efforts. The M2W component has strengthened the revolutionary CLTS approach by providing real-time monitoring and feedback and reinforcing the drive toward ODF status.
